# Treating Patients With ANCA-Associated Vasculitis and Very Severe Renal Injury With an Intensified B Cell Depletion Therapy: Comparison With a Control Cohort Receiving a Conventional Therapy

**DOI:** 10.3389/fimmu.2022.777134

**Published:** 2022-03-24

**Authors:** Dario Roccatello, Savino Sciascia, Stefano Murgia, Giacomo Quattrocchio, Michela Ferro, Emanuele De Simone, Carla Naretto, Antonella Barreca, Andrea Sammartino, Daniela Rossi, Roberta Fenoglio

**Affiliations:** ^1^ University Center of Excellence on Nephrologic, Rheumatologic and Rare Diseases (ERK-net, ERN-Reconnect and RITA-ERN Member) with Nephrology and Dialysis Unit and Center of Immuno-Rheumatology and Rare Diseases (CMID), Coordinating Center of the Interregional Network for Rare Diseases of Piedmont and Aosta Valley, San Giovanni Bosco Hub Hospital, Torino, Italy; ^2^ Department of Clinical and Biological Sciences of the University of Turin, Torino, Italy; ^3^ Pathology Unit, Città della Salute e della Scienza, Torino, Italy

**Keywords:** ANCA-associated vasculitis, rituximab, glomerulonephritis, vasculitis, polyangiitis and granulomatosis, polyangiitis

## Abstract

Rituximab (RTX), an anti-CD20 monoclonal antibody, has shown to be an effective induction treatment for small-vessel vasculitides associated with antineutrophil cytoplasm antibodies (AAV) in both newly diagnosed and relapsing patients. However, the role of RTX in the management of the most severe cases of AAV remains to be fully elucidated. The aim of this study was to assess both safety and efficacy of an intensified B-cell depletion therapy (IBCDT) protocol, including RTX, cyclophosphamide (CYC), and methylprednisolone pulses without additional maintenance immunosuppressive therapy in a cohort of 15 AAV patients with the most severe features of AVV renal involvement (as <15 ml/min GFR and histological findings of paucimmune necrotizing glomerulonephritis with more than 50% crescents of non-sclerotic glomeruli at the renal biopsy). Results of the IBCDT regimen have been compared to those obtained in a control cohort of 10 patients with AAV treated with a conventional therapy regimen based on oral CYC and steroids followed by a prolonged maintenance therapy with azathioprine (AZA). Plasma exchange was equally employed in the study and the control group. Complete clinical remission (BVAS 0) was observed at 6 months in 14 of 15 patients treated with IBCDT (93%). All cases who achieved a complete clinical remission experienced a depletion of peripheral blood B cells at the end of therapy. Of the 10 dialysis dependent patients at onset, 6 subjects (60%) experienced a functional recovery allowing the suspension of dialysis treatment. When compared to the control group, no statistically significant difference was observed in patients treated with IBCDT in terms of overall survival, 6-month therapeutic response rate, and 6-, and 12-month functional renal recovery. The cumulative total dose of CYC in the case group was on average 1 g/patient while in the control group on average 8.5 g/patient (p = 0.00008). Despite the retrospective design and relative limited sample size, IBCDT appeared to be safe and had the same efficacy profile when compared to the conventional therapy with CYC plus AZA in the management of the most severe patients with AAV. Additionally, this avoided the need of prolonged maintenance therapy for long, and limited the exposure to CYC with consequent reduced toxicity and drug-related side effect rates.

## Introduction

Granulomatosis with polyangiitis (GPA) and microscopic polyangiitis (MPA) are small vessel vasculitides associated with antineutrophil cytoplasm antibodies (ANCA) (1). Classification has been historically based on the clinical phenotype together with ANCA positivity or histological confirmation (2). More recently, a genome-wide association (GWAS) study showed that, from a genetic perspective, ANCA-associated vasculitides (AAV) are best defined by ANCA specificity rather than clinical aspects (3). Disease severity is heterogenous and ranges from mild features to life- or organ-threatening manifestations, especially in the proteinase 3 (PR3)-associated group (4). AAV have a chronic relapsing course leading to prolonged exposure to immunosuppression with increased toxicity, drug-related side effects, and organ damage (5).

The therapeutic options have grown over the years with the aim of decreasing the adverse effects, improving the quality of life, and maintaining the disease under complete control. Biotechnological approaches represent the possibility to act on diverse pathogenic pathways and personalize the management. Among the others, when considering both newly diagnosed and relapsing AAV patients, Rituximab (RTX) has been shown to be an effective induction treatment (6–8). A growing body of evidence, from both retrospective (9, 10) and prospective studies (10), has explored the efficacy of repeat dose RTX as maintenance therapy. Nevertheless, a definitive answer still needs to be provided, including the number/yearly and amounts of repeat dose RTX, and duration of maintenance treatment (11). Whether or not, in maintenance therapy, re-dosing should be given at a fixed time interval or driven by rising of ANCA titre and B cell count or symptoms, or both, has been addressed in a randomized controlled study (12), but is still under discussion. Moreover, the role of RTX in the management of the most severe cases of AAV with still florid paucimmune necrotizing glomerulonephritis remain to be fully elucidated. The concept of *severe* paucimmmune necrotizing glomerulonephritis has been used to define the cases with either <50 ml/min GFR (PEXIVAS, RAVE trials) or <30 ml/min or GFR (MEPEX, RITUXVAS trials). However, those with <15 ml/min/1.73m^2^ GFR, especially if dialysis-dependent, are undoubtfully the most severe and challenging niche of patients. Evidence of effective treatment in this specific subset of patients are lacking.

We previously investigated the promising effects in patients with severe autoimmune conditions, who were treated with an intensified B-cell depletion therapy (IBCDT) protocol, including RTX, cyclophosphamide (CYC), and methylprednisolone pulses (13–16). Safety and efficacy of the IBCDT scheme have already been proven both for induction of remission and long-term remission maintenance.

The aim of this study was to assess both safety and efficacy of the IBCDT protocol in a cohort of biopsy proven AAV patients with the most severe features of renal involvement when compared with a control group of patients with AAV treated with a conventional therapy regimen based on CYC/AZA and steroids.

## Materials and Methods

### Design of the Study and Inclusion Criteria

This is a retrospective, single-center open-label study that included all consecutive patients diagnosed with AAV (1) and treated with IBCDT from 2012-2020 meeting the following inclusion criteria:

a) severe renal injury defined as <15 ml/min/1.73m^2^GFR *and* histological findings of paucimmune necrotizing glomerulonephritis with more than 50% crescents of non-sclerotic glomeruli at the renal biopsy;
b) At least one year of follow-up after the IBCDT.

Controls were selected among the cohort of patients being treated for similarly severe AAV with renal involvement at the Center and were matched based on histology, ANCA-profile, age, and indication for treatment (newly diagnosed, new relapse or refractory renal disease).

### Protocols

IBCDT: RTX 375 mg/m^2^ administered for four weekly doses (on days 1, 8, 15, and 22), followed by two more doses (375 mg/m^2^) after 30 and 60 days, respectively. Additionally, two administrations of 10 mg/kg of intravenous CYC (reduced by 30 to 50% according to renal impairment) at days 4 and 17, plus three methylprednisolone pulses (15 mg/kg) at days 1, 4, and 8, subsequently followed by oral prednisone according to the following tapering scheme: 1 mg/kg/day for two weeks, 0.75 mg/kg/day, 0.5 mg/kg/day for another one month, followed by tapering by 5 mg each every fortnight until 5 mg/day to be reached within the sixth month and then discontinued. No further immunosuppressive maintenance therapy is administered.

CD19+B-cells and ANCA have been strictly monitored. In the case of CD19+ cells re-population *and* ANCA increase (defined as *biochemical relapse*), a maintenance regimen of RTX 500 mg every 4 months for 2 years followed by 500 mg every 6 months for 1 year was established.

A re-induction therapy with 4 RTX infusions of 375 mg/m^2^ weekly (instead of 6), together with a faster tapering of oral corticosteroids aimed to discontinue the drug within the third month, has been reserved to the case of overt clinical relapse. In the case of re-induction, a maintenance regimen of RTX 500 mg every 4 months for 2 years, followed by 500 mg every 6 months for 1 year, was planned.

Control patients received an induction therapy with oral CYC at the dose of 2 mg/kg/day for 3-6 months and three intravenous pulses of methylprednisolone of 15mg/kg (max 1000 mg) on three consecutive days, followed by an oral dose of prednisone 1mg/kg/day (max. 60mg/day) to be tapered to 12.5 mg at the end of month 3 and to 5 mg at month 18, and a maintenance immunosuppressive therapy with oral azathioprine (2 mg/kg/day for at least 2 years and 1-1.5 mg/kg/day for additionally 1-2 years).

Independently on the pharmacologic regimen, plasma exchange (7 procedures with 1-1.5 plasma volume replacement) was performed in the presence of 1. alveolar haemorrhage, together with more than 50% florid crescents in the non-sclerotic glomeruli, or 2 dialysis dependence.

Plasmapheresis sessions were performed at part of the induction therapy among 13 patients (87%) in the case group and 8 (80%) in the control group.

### Assessment of Renal Function

The Chronic Kidney Disease Epidemiology Collaboration equation (17) was assessed to measure eGFR.

### Response to Treatment

The Birmingham Vasculitis Activity Score for Wegener Granulomatosis (BVAS v3.0) (18) has been calculated to quantify disease activity at study entry, monthly for the first 6 months and every six months during the follow-up or in case of relapse. Remission was defined by a BVAS/WG of 0.

Response was evaluated by assessing the changes in clinical signs and symptoms and laboratory parameters. Renal remission was defined as stabilization or improvement of eGFR together with improvement of hematuria (<10 erythrocytes per high power microscopic field) and proteinuria (<1 g/24 hours). Relapse was defined as reappearance or worsening of disease as measured with an increase of at least 1 point of BVAS when compared to the score value obtained after previous treatment and involvement of at least one major organ.

Biochemical relapse was defined as re-appearance of ANCA together with CD19+ cell re-population in the absence of overt clinical symptoms.

Adverse events and subsequent complications have been reported. Severe infections were defined as those requiring hospitalization or intravenous antimicrobial therapy.

A subgroup of patients in both groups who agreed to the procedure underwent a second “protocol” renal biopsy 6-12 months following the beginning of induction therapy.

### Statistical Analysis

For comparison of variables at baseline and follow-up, Student’s t-test for normally distributed parameters and Mann Whitney’s test for non-normally distributed parameters, Fisher’s test for categorical variables, were used as appropriate. Univariate Descriptive Statistics was used to identify any predictors of AAV relapse. For these analyses, the SPSS (IBM Corporation, NY, USA) software program was used. p < 0.05 was considered significant.

This study was conducted according to the Piedmont and Aosta Valley (North West Italy) legislation for Rare Diseases (N. 1577/UC/SAN of 11.10.2005 based on Regional Government Act 23 April 2007 dealing with Rare Diseases, Systemic Sclerosis RM0091; article. 1: 796 paragraph Z Law number 296 of 2006. Number 5-5740). The study was conducted according to the Helsinki Declaration and each patient provided written consent to participate.

## Results

Fifteen consecutive patients with AAV who met the inclusion criteria were deemed eligible for IBCDT. A second group, representing the control group, consisted of ten consecutive patients with AAV, and with comparable clinical and histological characteristics who received induction therapy with CYC. Patients were given one of the two treatment regimens, according to her/his choice, after being informed of the off-label nature of the experimental treatment. Baseline demographic and clinical-laboratory parameters are shown in **Table 1**. The median age of the case studies (8 men; 7 women) was 69 ± 11.6 years (39-86 years) while the median age of controls (7 men; 3 women) was 72 ± 12.4 years (50 - 86 years).

**Table 1:** Baseline demographic and clinical characteristics of the study and control group.

Parameter	IBCDT(n 15)	CYC(n 10)	p value
**Age [(median)range]**	71 (49-86)	72 (50-89)	0.46
**M/F**	8/7	7/3	0.23
**MPA/GPA**	11/4	8/2	0.25
**MPO/PR3**	11/4	7/3	0.85
**BVAS median [(median)range]**	21 (11-30)	23 (12-30)	0.54
**N° organ/system involvement [(median)]**	3	3	>0.99
**sCr [(median)range]**	8 (3.9-20)	8.4 (4.3-22)	0.85
**eGFR [(median)range]**	9 (7-15)	9.5 (7-15)	0.79
**HD (%)**	10 (66)	6 (60)	0.73
**Florid crescentic epithelial (%) [(median)range]**	57 (51-66)	52 (51-69)	0.37
**Glomerulosclerosis (%) [(median)range]**	21 (9-27)	20 (11-26)	0.88

IBCDT,intensified protocol of B lymphocyte depletion; CYC, cyclophosphamide; MPA, Micropolyangiitis; GPA, granulomatosis with polyangiitis; MPO, myeloperoxidase; PR3, Leukocyte proteinase 3; BVAS, Birmingham Vasculitis Activity Score; SD, standard deviation; sCr, serum creatinine; eGFR, estimated glomerular filtration rate (CKD-Epi); HD, haemodialysis dependency.

No statistically significant differences were observed when comparing baseline demographics and laboratory-instrumental characteristics of cases and controls.

The median BVAS at onset in the case group was 21 [range 11-30] and in the control group 23 [range 12-30]. Ten patients in the case group (66%) and 6 patients in the control group (60%) required dialysis at onset.

All patients in both groups had histologically proven renal involvement at onset as per inclusion criteria. Patients of the study group presented a median of 57% (51-66) of epithelial/fibroepithelial crescents on the total of non-sclerotic glomeruli and 21% of global glomerulosclerosis; in the control group, median was 52% of epithelial/fibroepithelial crescents with 20% of global glomerulosclerosis.

At baseline, 12 patients (80%) in the study group presented pulmonary involvement as follows: 7 (47%) alveolar haemorrhage, 3 (20%) fibrosing interstitial disease, 5 (33%) pulmonary nodularities. In the control group, 7 patients (70%) had lung involvement, 4 (40%) with alveolar haemorrhage 3 (30%) with fibrosing interstitial disease and 3 (30%) with pulmonary nodularity. Up to a third of the patients presented with a multiple lung injuries pattern.

### Therapeutic Response

In the cases treated with IBCDT, complete clinical remission (BVAS 0) was observed at 6 months in 14 of 15 patients (93%) in all cases being associated with complete depletion of peripheral blood B cells at the end of therapy. In detail, all patients had complete peripheral blood-B cell depletion after the first “4+2” RTX protocol. The CD19+ B cells were detectable in the circulation after a mean of 13.6 months (9-20 months).

There were no infusion reactions or early side effects attributable to the RTX, such as having to interrupt the therapeutic cycle.

Of the 10 dialysis dependent patients at onset, 6 subjects (60%) experienced a functional recovery allowing the discontinuation of dialysis treatment. The remaining 4 continued the replacement therapy. A statistically significant reduction in median sCr from 4.3(3.9-5.2) mg/dL at baseline to 1.9 (1.2-4.4) mg/dL at 6 months and 1.6(1.1-4.4) mg/dL at 12 months was observed in the non-dialysis depended patients (**Table 2**).

**Table 2:** Case group parameters at baseline before and after 6 months of IBCDT.

BASELINE							6 MONTHS				
PzIBCDT	ANCA	BVAS	sCr	HD	PEX	Lung	BVAS	sCr	ESRD	EXITUS	Lung recovery
**Pz 1**	PR3	20	4.3	NO	NO	NO	0	1.9	NO	NO	NA
**Pz 2**	MPO	21	6.5	YES	NO^#^	NO	0	NA	YES	NO	NA
**Pz 3**	MPO	19	4.4	YES	YES	NO	0	4.9	NO	NO	NA
**Pz 4**	PR3	25	7	YES	YES	YES (A)	0	NA	YES	NO	IM
**Pz 5**	MPO	21	20	YES	YES	YES (I; A; N)	0	NA	YES	NO	M
**Pz 6**	MPO	18	6.3	YES	YES	NO	0	3.5	NO	NO	NA
**Pz 7**	MPO	30	4	NO	YES	YES (A)	0	1.2	NO	YES	NA
**Pz 8**	MPO	18	8.1	YES	YES	YES (A)	0	NA	YES	NO	R
**Pz 9**	MPO	21	9	YES	YES	YES (I)	0	1.3	NO	NO	IM
**Pz 10**	MPO	27	4.9	YES	YES	YES (I; A)	0	1.2	NO	NO	S
**Pz 11**	PR3	22	7.9	YES	YES	YES (N)	0	2.4	NO	NO	R
**Pz 12**	MPO	20	5.2	NO	YES	YES (A)	6	4.4	YES	NO	R
**Pz 13**	MPO	12	8.8	YES	YES	NO	0	3.3	NO	NO	NA
**Pz 14**	MPO	26	4.4	NO	YES	YES (N; A)	0	2.3	NO	NO	R
**Pz 15**	PR3	25	3.9	NO	YES	YES (N; A)	0	1.4	NO	NO	R

PEX, plasmapheresis; N, pulmonary nodularity; I, interstitial disease; A, alveolar haemorrhage, NA, not applicable; R, total recovery; IM, improvement; S, stabilization. ^#^PEX limited to 3 sessions due to plasma intolerance.

In the controls, a complete response was observed at 6 months in 7 of 10 patients (70%) (**Table 3**). Two patients were switched to RTX-based therapeutic regimen due to persistent disease activity, after 6 and 7 months, respectively, from CYC induction. A third patient was switched to RTX regimen 6 months after CYC induction due to gastrointestinal intolerance. Three out of 6 dialysis dependent patients (50%) discontinued dialysis after induction with CYC. One dialysis dependent patient died 2 months after diseases onset. A statistically significant reduction in median sCr from 4.3(4.1-4.8) mg/dL at baseline to 2.75(1.5-4.5) mg/dL at 6 months and 1.7 (1.1-4.4) mg/dL at 12 months was observed in the non-dialysis depended patients.

**Table 3:** Control group parameters at baseline and at 6 months after induction with cyclophosphamide (CYC).

BASELINE							6 MONTHS				
Pz CYC	ANCA	BVAS	sCr	HD	PEX	Lung	BVAS	sCr	ESRD	EXITUS	Lung recovery
**Pz 1**	MPO	18	4.3	NO	YES	YES (I; A)	0	1.5	NO	NO	S
**Pz 2**	MPO	30	4.8	NO	NO	YES (I)	0	4.5	NO	NO	W
**Pz 3**	MPO +Ab GBM	16	7.8	YES	YES	NO	0	NA	YES	YES	NA
**Pz 4**	PR3	24	3.35	NO	NO	YES (A, N)	13	2.6	NO	NO	W
**Pz 5**	PR3	27	8.9	YES	YES	YES (A, N)	0	1.7	NO	NO	M
**Pz 6**	MPO	24	22	YES	YES	NO	19	4.9	NO	NO	NA
**Pz 7**	MPO	21	8.5	YES	YES	YES (I, N)	0	2.4	NO	NO	IM
**Pz 8**	MPO	28	6.4	YES	YES	YES	0	5.4	YES	NO	IM
**Pz 9**	PR3	12	11	YES	YES	NO	0	N/A	YES	NO	NA
**Pz 10**	MPO	22	4.1	NO	YES	YES (A)	10	2.9	NO	NO	S

PEX, plasmapheresis; N, pulmonary nodularity; I, interstitial disease; A,alveolar haemorrhage; NA, not applicable; R, total recovery; IM, improvement; S, stabilization; W, worsening.

### Follow Up

Patients treated with IBCDT were followed for a median of 44 months (27-98). Relapses (1 renal and 2 extra-renal) occurred in three patients after 12, 19 and 58 months respectively from onset. In all cases, relapses were preceded by an increase in the ANCA titre paralleled by CD19+ B lymphocyte repopulation.

The first patient, MPO positive, after 12 months showed constitutional symptoms, reappearance of micro-haematuria and increase in proteinuria up to 2 g/24h. After undergoing renal biopsy, histological lesions from active vasculitis with florid crescents were detected in 10% of non-sclerotic glomeruli.

The second patient was MPO positive with a clinical phenotypic presentation compatible with GPA (rhinosinusitis, finding of granulomatous inflammation on lung biopsy) after 19 months presented worsening of sinusitis, bilateral pulmonary thickening and urinary abnormalities; on renal biopsy, florid crescents were found on 27% of non-sclerotic glomeruli.

A third patient, PR3 positive, relapsed with scleritis, arthralgia, subcutaneous nodules and peripheral neuropathy.

All three patients were treated with a second cycle of RTX as per protocol with disappearance of symptoms and achieving a new complete remission by 3 months. They were started on maintenance therapy with RTX in two cases and enrolled in an experimental clinical trial in one case.

No biochemical relapse was observed.

We observed 3 cases of cytomegalovirus viraemia without invasive organ disease, 1 pulmonary aspergillosis, and 1 systemic candidiasis. No patient suffered for infections requiring hospitalization. In all cases it was possible to effectively control these complications with antimicrobial therapy. There were no deaths from infectious causes.

In the early phase of treatment there were 3 adverse effects attributable to steroid therapy: in 2 cases the onset of diabetes mellitus, 1 episode of psychiatric decompensation in a patient with known bipolar disorder.

No patient experienced a drop in IgG < 300 mg/dl (a level generally assumed as the cut-off value for IVIG replacement therapy). Accordingly, no patients in our cohort underwent immunoglobulin replacement. At 6 months, 7 (46%) presented with IgG level ranging between 300-500 mg/dl (mean 417 mg/dl, range 371-489) that persisted in the same range in 3patients (20%) at 12 months. At 18 months from the IBCDT, no patient presented with IgG level < 500 mg/dl.

There were no further adverse events attributable to steroid and immunosuppressive therapy.

A 83-year-old patient died 6 months after IBCDT for cardiovascular causes not attributable to vasculitis. Three responsive patients among those who underwent to the IBCDT agreed to perform a subsequent kidney biopsy after clinical remission was achieved. Second biopsies were performed on average 11 months after the first ones. No sign of active vasculitic lesions were found. The rate of glomerulosclerosis in these patients is shown in **Table 4**.

**Table 4:** Findings of repeated renal biopsy performed during clinical remission in the two groups.

1° BIOPSY	sCr	HD	Florid epitelial Crescent	Glomerulo Sclerosis (%)	2° BIOPSY	sCr	ESRD	Florid epithelial crescent	Glomerulo Sclerosis (%)
**PZ** **IBCDT1**	6.3	YES	25%	35%	**PZ IBCDT2**	3.3	NO	0%	69%
**PZ** **IBCDT2**	9.0	YES	35%	26%	**PZ IBCDT3**	1.5	NO	0%	22%
**PZ** **IBCDT3**	4.9	YES	36%	40%	**PZ IBCDT4**	1.8	NO	0%	56%
**PZ CYC1**	13.9	YES	47%	0%	**PZ CYC1**	1.9	NO	0%	66%
**PZ CYC2**	8.5	YES	69%	7%	**PZ CYC2**	1.9	NO	0%	32%
**PZ CYC3**	6.4	YES	62%	0%	**PZ CYC3**	NA	YES	0%	54%

HD, haemodialysis dependency; sCr, serum creatinine; IBCDT, intensified protocol of B lymphocyte depletion; CYC, cyclophosphamide.

When referring to the control group, patients were followed for a median of 81 months (range 36-146 months).

Three patients suffered for a renal relapse after 26, 26 and 60 months from induction respectively. In all three cases a renal biopsy was performed at the time of relapse, showing the presence of active inflammation with epithelial crescents in 20, 43, and 37% of non-sclerotic glomeruli, respectively. No isolated biochemical relapse was observed.

Severe infectious complications were observed in 4 patients (two cytomegalovirus viremias, 1 pulmonary aspergillosis and one case of severe H. Zoster infection), which required hospitalization and/or i.v. antimicrobial therapy.

During the follow-up the following events were recorded: 2 patients developed meta-steroid diabetes mellitus, 1 case of prostate cancer (cumulative dose CYC 18 g), 1 patient developed Kaposi’s sarcoma, 1 patient a severe osteoporosis in coxarthrosis requiring hip replacement. One patient suffered for deep vein thrombosis.

Four patients died, respectively 2,9,20 and 58 months after diseases onset. Causes of deaths were 2 thromboembolic events, 1 massive gastrointestinal bleeding, 1 cardiac arrest.

Three patients agreed to perform a second renal biopsies in conditions of clinical remission. No case presented with active vasculitic lesions. The percentages of glomerular sclerosis at the first and second biopsy are shown in **Table 4**.

**Figure 1:**
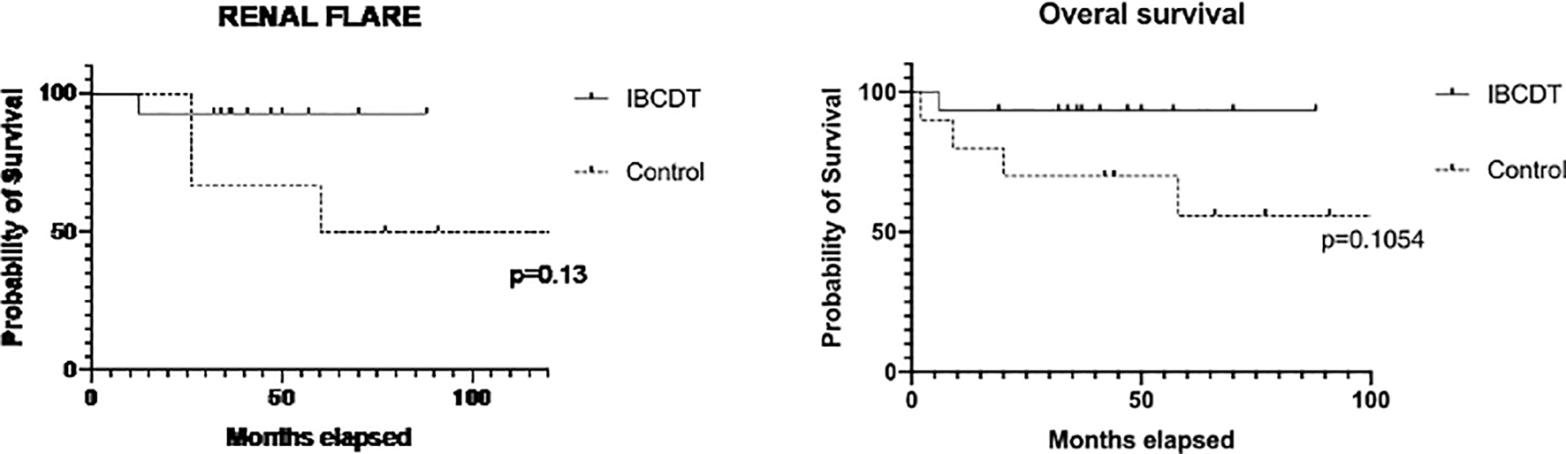
Time free from renal flares and overall survival (Kaplan–Meier curves) in IBCDT-treated patients and controls. IBCDT, intensified B-cell depletion induction therapy. Renal flares were evaluated excluding patients who died during the follow-up.

When compared to the control group, no statistically significant difference was observed in patients treated with IBCDT in terms of overall survival, 6-month therapeutic response rate, and 6-, and 12-month functional renal recovery. No difference between cases and controls was similarly observed when stratifying for ANCA profile (**Figure 1**).

The median duration of follow-up was 44 months (27-98) in the case group and 81 months (range 36-146 months) in the control group, respectively.

When pooling together cases and controls, we observed that diagnosis of MPA was associated with a trend in higher mortality at 6 months (2/19 Vs. 0/6). However, we failed to identify any further predictive factors. This may be an aspect related to the sample size.

**Figure 2:**
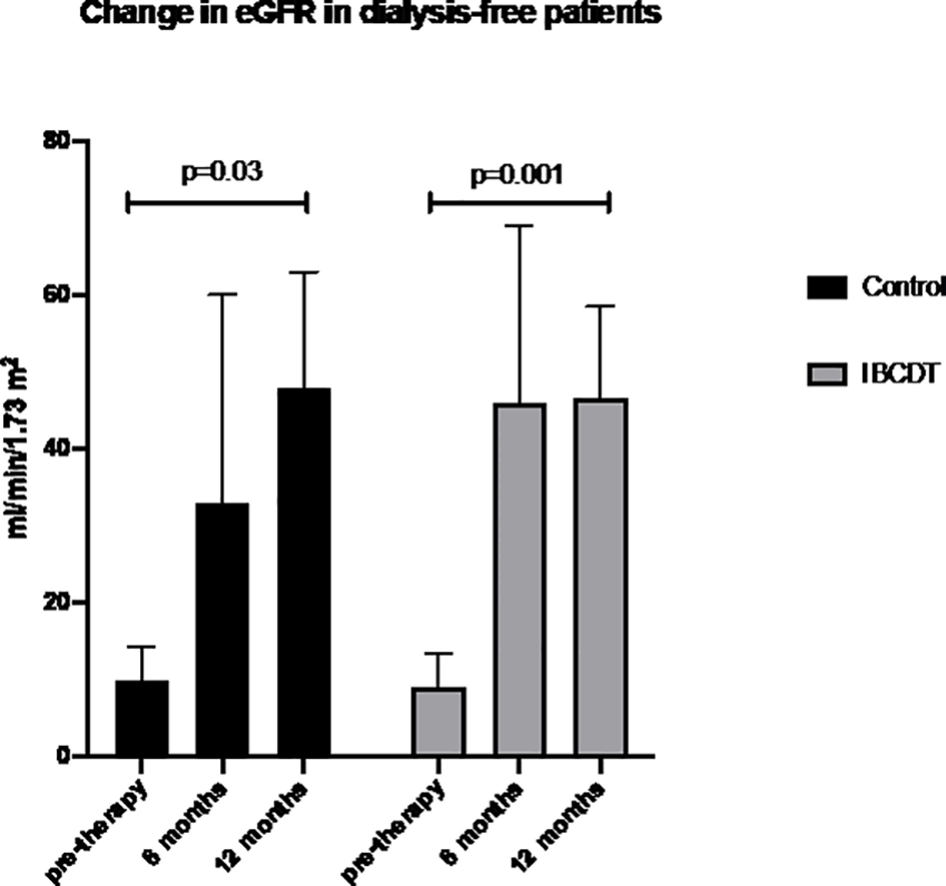
Sequential eGFR for the IBCDT and daily oral cyclophosphamide groups in pa dialysis-free patients. Data are presented as mean ± SD. eGFR = estimated glomerular filtration rate. IBCDT, intensified B-cell depletion induction therapy.

In dialysis-free patients treated with IBCDT we observed a statistically significant improvement in eGFR (CKD-Epi) from 9 ml/min to 46 ml/min at 6 months (p = 0.001) and 46.5 ml/min at 12 months. Similarly, a significant increase in eGFR from 10 mL/min to 33 mL/min at 6 months (p = 0.03) and 48 mL/min at 12 months was observed in CYC-treated dialysis-free patients (**Figure 2**).

During the follow-up, we observed a higher rate of very severe infections in the CYC group when compared to IBCDT group (0/15 Vs. 4/10), resulting in one severe infection for every 16.7 patient years in the study in the control group compared to no severe infection among patients treated with the IBCDT (cumulative years of observation 63 years).

Prophylaxis with trimethoprim/sulfamethoxazole for *pneumocystis carinii pneumonia* was given in 13 patients (86%) in the case group and 7 (70%) in the control group.

There was a similar number of relapses in the two treatment groups (3 events for each patient group).

In both groups, repeated renal biopsies performed during clinical remission (**Table 4**) did not show any active vasculitic lesions, while a trend towards a relative greater increase in the percentage of glomerulosclerosis was observed in the group treated with CYC.

The cumulative total dose of cyclophosphamide in the case group was on average 1 g/patient while in the control group on average 8.5 g/patient, a difference that was statistically significant between the two groups (p = 0.00008). Calculated on a monthly basis, and adjusted for follow-up, the IBCDT regimen corresponds to a mean reduction in the cumulative dose of CYC equivalent to 836 mg/month.

A comparison of overall safety and efficacy profiles at the end of follow-upare shown in **Table 5**.

**Table 5:** Comparison of overall safety and efficacy profile at the end of follow-up.

	IBCDT(n=15)	Controls(N=10)	p
Relapse (any)	3	3	0.65
Renal Relapse	1	3	0.27
Deaths	1	4	0.12
Side effects			
Severe Infections (hospitalization and/or i.v. treatment)	0	4	0.02
Steroid-induced diabetes	2	1	0.99
Psychiatric episode	1	0	0.99
Osteoporosis	0	1	0.40
Glaucoma	1	3	0.27

## Discussion

ANCA-associated vasculitis can be characterized by a severe acute onset and, especially in the PR3-ANCA positive form, a chronic recurrent course. A prompt control of the vascular inflammatory process is necessary to prevent the onset of irreversible and potentially fatal damage to organs. Similarly, preventing relapses limits organ damage and excessive exposure to steroids and immunosuppressants, which results in severe infections and malignancy (especially urological and haematological) in the elderly, and infertility in younger patients.

RTX has been extensively studied in numerous observational reports (6-11). In two randomized controlled trials, it was proven to be at least as effective as CYC in inducing remission in newly diagnosed AAV patients (6, 7). RTX was found to be superior to CYC in the management of relapsing diseases (7).

Recently, efficacy of RTX compared to CYC in AAV patients was examined in a Mayo Clinic observational study with the largest number of patients with eGFR<30 ml/min per 1.73 m^2^ even reported (19). No statistically significant differences between RTX and CYC for remission induction therapy was found, neither an apparent benefit from the addition of plasma exchange. Treatment did not follow a strictly standardized protocol, and a separate analysis of patients who needed dialysis at baseline was not provided.

We have previously shown a favourable outcome in patients with refractory AAV treated with a “4+2” RTX protocol (20). RTX was administered after unsuccessful or not tolerated cycle(s) of cyclophosphamide in 9 out 11 patients. The “4+2” protocol (so called “improved protocol”) (20) had been generally well tolerated, but its long-term safety profile in AAV remains to be demonstrated.

The aim of this study was to evaluate the efficacy and safety of an “intensified” scheme combining the RTX improved protocol (4 + 2 infusions) and low doses of cyclophosphamide in AAV patients with very severe renal involvement, i.e., <15 ml/min eGFR per 1.73 m^2^. Efficacy has been measured by combining clinical, laboratory and histological parameters. Results were compared to those achieved by a conventional scheme of CYC-AZA in a control cohort of similarly severe cases.

One single cycle of IBCDT was proven to be comparable to CYC induction therapy followed by maintenance with azathioprine. In detail, we observed complete remission at 6 months in up to 95% of the cases treated with IBCDT. The absence of signs/symptoms of active vasculitis, and even the renal histological features, confirmed the effectiveness of the treatment. In our series, almost all of the patients treated with the intensified B lymphocyte depletion protocol achieved a prednisone dose <5 mg/day or discontinued the steroid completely within 6 months. IBCDT was associated with a lasting remission, avoiding subsequent maintenance immunosuppressive therapy in most patients.

The novelty aspects of our studies required some consideration.

First, the peculiar sample of cases under study. The severity of renal involvement, i.e., extensive extracapillary necrotizing glomerulonephritis, was histologically documented and up to 67% of patients needed dialysis at the onset. Outcome data from patients with aggressive AAV with very severe renal involvement are poor and, as expected on prevalence basis, are derived from a small series often shuffled in larger cohorts (6, 21, 22). Data are confounding when mixed in heterogeneous patient samples, and results of therapy are difficult to interpret. Our study demonstrates the effectiveness and the safety of an aggressive immunologic intervention in these very severe patients with AAV, a category that should be probably identified apart in future studies.

Secondly, the combined approach with low dose CYC and RTX. The goal of this study was to show that complete remission can be obtained reducing the cumulative dose of CYC and the incidence of adverse effects. Although the complete remission was achieved in both groups of patients, in the control group a median cumulative CYC dose of 8.5 g/patient was used, while the group treated with the IBCDT was exposed to a median cumulative CYC dose of only 1g/patient. The rationale for adding a low dose of CYC to induction lies in the intrinsic pharmacological properties of the RTX and in the cumulative effects of the two drugs on lymphocytes and B cell depletion. The synergistic use of a low dose of CYC allows quicker action on the pathogenic cell lines, potentially increasing the rapidity of response, and reducing the cumulative dose of alkylating agents.

Third, the extent of efficacy. In our series, 95% of the patients treated with the intensified B lymphocyte depletion protocol achieved a prednisone dose 5 mg/day or discontinued the steroid completely within 6 months. This rapid tapering resulted in a relatively low rate of steroid-related side effects. Our observations are in line with what was observed in a *post-hoc* analysis of the patients with <30 ml/min per 1.73 m^2^ eGFR in the RAVE study (23). In the randomized controlled trial RITUXVAS (6) the lymphoma RTX protocol plus 2 CYC administrations was used in AAV patients with more severe renal involvement than RAVE trial. Complete remission was observed in 25 of 33 patients (76%). When considering only the surviving patients, a complete response was obtained in 93% of cases. Using a more intensive RTX regimen, adopting the same definition of remission and treating patients with severe multiorgan compromise, we observed a complete response in 14 out of 15 patients (93%). In our case series, 10 patients were dialysis dependent at onset and 60% of them experienced at least a partial recovery of their renal function after IBCDT.

A further level of novelty relies on the possibility of avoiding maintenance therapy (especially in MPO-AAV). Up to 85% of our patients treated with a single therapeutic cycle of IBCDT had a relapse-free course on a median follow-up of 44 months (27-98). This observation supports the concept that a maintenance immunosuppressive therapy is not indiscriminately needed in all cases, especially in MPO-AAV patients who are less susceptible to relapse. A “watchful waiting” approach, with a serial monitoring of the ANCA titres and CD19+ cells, might be reasonable in these cases, albeit the role of CD19+ cells *per se* in guiding therapeutic choices might need further confirmation (24). Additionally, we observed a trend for a higher incidence of deaths (albeit not statistically significant) and a higher rate of steroid and immunosuppression-related side effects in the group treated with CYC compared to IBCDT. Of course, the relatively small sample-size imposes caution when interpreting these results. Nevertheless, the higher exposure to side effects in our control group is in line with the results of the CYCLOPS study, where the use of i.v. cyclophosphamide was compared to oral cyclophosphamide (25).

When referring to the histological aspects, reports of repeat renal biopsies in patients with AAV are rare in the literature. In 3 patients treated with IBCDT, repeat renal biopsies were performed in clinical remission, on average one year after onset and in the absence of maintenance therapy. No histological signs of active disease were observed. As expected, an increase in glomerular sclerosis was found on average from 33.6% to 49% of the glomeruli. These results are consonant with those of a monocentric experience on AAV patients with renal involvement who underwent protocol renal biopsies (26). Incidentally, in our control patients treated with CYC-AZA who underwent a second biopsy, in addition to the disappearance of crescents and necrotizing lesions, a relatively higher rate of glomerulosclerosis was noticed when comparing the first with second biopsies (on average from 2.3 to 50% of the glomeruli).

Most patients in our study underwent plasmapheresis sessions as part of the induction therapy. As per protocol, PLEX was equally distributed in the two groups. When referring to the added value of combining PLEX with IBCDT, a synergic effect cannot be excluded. A multicenter study (27) retrospectively analysed the outcome of 129 patients with AAV treated with a combination therapy of RTX and a reduced dose of oral CYC plus PLEX in cases of rapidly progressive renal failure or alveolar haemorrhage. At 6 months, 91% of patients achieved complete remission with no significant difference between MPO and PR3 positive patients. At one year of follow-up under maintenance therapy with RTX every 4 months, a total of 6 relapses (5 minor) were observed. Results of PEXIVAS trial (28) seemed to indicate that PLEX did not reduce the risk of end stage kidney disease in patients with AAV. This study included patients with eGFR<50 ml/min per 1.73 m^2^, with evidence of pulmonary haemorrhage only in one third of cases, while hemodialysis was required in one fifth of cases. Inclusion of a substantial proportion of patients with mild disease might have obscured the detection of benefits for the most critical cases. Kidney biopsy was not required for entry into the study so that the extent of chronic renal lesions could not be determined. Patients with advanced fibrosis would not be expected to improve with any treatment, including PLEX. The study also lacked stratification aimed at identifying the cases with very reduced renal function, who, instead, were the main focus of our study. Another limitation of the PEXIVAS trial which generally goes unnoticed is that the effect of medical treatment in patients given RTX had probably been diminished when PLEX had been performed before the fifth day following RTX. Due to RTX kinetics, a shorter interval might have caused a considerable removal of RTX.

Our study has some main limitations, such as lack of randomization, its retrospective nature and the relatively small size of the samples. Besides, IBCDT was not compared with RTX given alone at standard doses nor other regimens besides CYC+AZA. However, our patients were more severe than those of RAVE trial in which the standard doses of RTX were used. And, when compared to RITUXIVAS study, which included patients as severe as our cohort but used a less intensive scheme, IBCDT ensured a disease-free survival in most patients without an immunosuppressive maintenance regimen over a longer follow-up.

The limitations of the present study are offset by the inclusion of patients of advanced age, with multiple co-morbidities and with severe renal impairment, which are representative of the real-life AAV population.

In conclusion, the results of this study showed that IBCDT was safe and had the same efficacy profile of a conventional therapy with CYC plus AZA in the induction of remission of the most severe patients with AAV. In addition, IBCDT avoided the need for maintenance therapy in the long term, and reduced the exposure to CYC.

Who could especially benefit from this regimen? The best candidates are the patients with diffuse crescentic necrotizing glomerulonephritis and very rapidly progressive course with or without lung involvement who are approaching or require dialysis.

## Data Availability Statement

The raw data supporting the conclusions of this article will be made available by the authors, without undue reservation.

## Ethics Statement

Ethical review and approval was not required for the study on human participants in accordance with the local legislation and institutional requirements. The patients/participants provided their written informed consent to participate in this study.

## Author Contributions

DRoc, SS, designed the study, data analysis and drafted the manuscript. SM, GQ, MF, ED, CN, AB, AS, DRos, and RF, were involved in data collection, patients follow-up, data review, and critically reviewed the draft. All authors contributed to the article and approved the submitted version.

## Conflict of Interest

The authors declare that the research was conducted in the absence of any commercial or financial relationships that could be construed as a potential conflict of interest.

## Publisher’s Note

All claims expressed in this article are solely those of the authors and do not necessarily represent those of their affiliated organizations, or those of the publisher, the editors and the reviewers. Any product that may be evaluated in this article, or claim that may be made by its manufacturer, is not guaranteed or endorsed by the publisher.
